# The impact of elective spine surgery in Canada for degenerative conditions on patient reported health-related quality of life outcomes

**DOI:** 10.1038/s41598-025-03613-4

**Published:** 2025-05-31

**Authors:** Ragavan Manoharan, Nisaharan Srikandarajah, Jean-Christophe Murray, Christopher Nielsen, Supriya Singh, Sean Christie, Michael M.H. Yang, Michael Weber, Bernard Larue, Adrienne Kelly, Jerome Paquet, Raphaele Charest-Morin, Guy Hogan, Andrew Glennie, Henry Anh, Eugene Wai, Nicolas Dea, Neil Mason, Kenneth Thomas, Charles Fisher, Hamilton Hall, Christopher Bailey, Mayilee Canizares, Yoga Raja Rampersaud

**Affiliations:** 1 University of Toronto Spine Program, Toronto Western Hospital, Toronto, ON Canada; 2 Department of Neurosurgery, Royal North Shore Hospital, Sydney, Australia; 3 Department of Neurosurgery, The Walton Centre, Liverpool, United Kingdom; 4 Faculty of Health and Life Sciences, University of Liverpool, Liverpool, England; 5 Division of Orthopaedic Surgery, Department of Surgery, CHU de Québec - Université Laval Quebec City, Quebec, QC Canada; 6 Schroeder Arthritis Institute, Krembil Research Institute, Department of Surgery, Division of Orthopaedic Surgery, University Health Network, University of Toronto, Orthopaedics, Toronto, ON Canada; 7 London Health Science Centre Combined Orthopaedic and Neurosurgery Spine Program, Schulich School of Medicine, Western University, London, ON Canada; 8 Department of Surgery, Division of Neurosurgery, Dalhousie University, Halifax, NS Canada; 9 University of Calgary Spine Program, University of Calgary, Calgary, AB Canada; 10 Department of Surgery, Division of Orthopaedics, Montreal General Hospital, McGill University, Montreal, QC Canada; 11 Département de Chirurgie, Faculté de Médecine et des Sciences de la Santé, Université de Sherbrooke, Sherbrooke, Québec Canada; 12 Sault Area Hospital, Northern Ontario School of Medicine, Sault Ste Marie, Sault Ste. Marie, ON Canada; 13 Centre de Recherche CHU de Quebec, CHU de Quebec-Universite Laval, Quebec City, QC Canada; 14 Combined Neurosurgical and Orthopedic Spine Program, Department of Orthopedics Surgery, University of British Columbia, Vancouver, BC Canada; 15 Department of Orthopaedic Surgery, Health Sciences Centre, Newfoundland and Labrador, St. John’s, Canada; 16 Department of Surgery, Division of Orthopedics, Dalhousie University, Halifax, NS Canada; 17 Division of Orthopaedic Surgery, St. Michael’s Hospital, Toronto, ON Canada; 18 Division of Orthopaedic Surgery, University of Ottawa, The Ottawa Hospital, Ottawa, ON Canada; 19 Canada East Spine Centre, Division of Orthopaedic Surgery, Horizon Health Network, Zone 2, Saint John, New Brunswick, Canada; 20 Dalhousie University Faculty of Medicine, Halifax, NS Canada; 21 Department of Surgery, University of Toronto, Toronto, ON Canada; 22 Schroeder Arthritis Institute, Krembil Research Institute, University Health Network, Toronto, ON Canada

**Keywords:** Spine surgery, Degenerative spinal conditions, Spinal health-related quality of life outcomes, Quality of life, Outcomes research

## Abstract

The impact of spine surgery on Health-Related Quality-of-Life (HRQoL) outcomes across common spinal degenerative diagnoses is not well characterised. A prospective observational study of patients enrolled in the Canadian Spine Outcomes and Research Network (CSORN) registry was performed. Baseline and 1-year post-operative Short Form-12 Physical Component Summary (PCS) and Mental Component Summary (MCS) scores were collated and compared to normative values from the Canadian General Population (CGP). The percentage of patients achieving the PCS Minimum Clinically Important Difference (MCID) was quantified. 5049 patients were included in the analysis. The mean pre-operative SF-12 PCS was 29.5 and MCS was 44.1. This improved to a mean PCS of 40.5 (*p* < 0.001) and MCS of 49.3 (*p* < 0.0001) at 1-year post-operatively. The mean pre-operative PCS was over 2 standard deviations (SD) lower than the normative mean of the CGP; this improved to being close to 1-SD from the normative CGP mean at 1-year post-operatively. Findings were similar across age- and sex-stratified subgroups. Across all conditions, 70–75% of patients achieved the PCS MCID. Fewer patients with cervical myelopathy achieved the PCS MCID (59%). In a surgical cohort, patients with degenerative spinal conditions demonstrate a profound reduction in PCS compared to their peers in the CGP. Spinal surgery was impactful in improving physical function HRQoL outcomes in the majority, but not typically to average population norms.

**Supplementary Information:**

The online version contains supplementary material available at 10.1038/s41598-025-03613-4.

## Introduction

Musculoskeletal disorders including joint osteoarthritis and low back pain are the leading cause of years lived with disability and have a significant impact on Health-Related Quality of Life (HRQoL) and functional capacity^1,2^. In the United States, symptomatic degenerative spinal conditions have become the most common cause of reduced HRQoL, with approximately 33 million patients requiring treatment for spine related issues^3^. This societal burden of degenerative spinal conditions is increasing as the population ages^4,5^. Correspondingly, the rate and associated cost of spine surgery has also increased to varying degrees across different countries^6,7^. The rising rate and associated cost of spinal surgery compared to its risks and benefits continues to draw scrutiny regarding appropriateness and effectiveness^6,7^.

In a resource limited environment, it is crucial to quantify the impact of an intervention to ensure appropriate societal investment. This validation has occurred in total hip and knee arthroplasty where societal demand, sustainable cost and clinical effectiveness have led to these procedures being accepted by all stakeholders (patients, health care professionals and providers, payers and government bodies)^8^. In limited studies, Rampersaud et al., has previously shown that surgical management of a sub-population of surgical spine patients with lumbar spinal stenosis with or without degenerative lumbar spondylolisthesis results in a similar and sustainable improvement of HRQoL compared to total knee replacement following surgery^9–12^. However, the overall impact of spine surgery on HRQoL outcomes for degenerative spinal disorders has not been well characterized in a national patient sample across the most common degenerative pathologies undergoing surgery.

Patient reported outcomes measures (PROMs) are commonly used to report the impact of surgery. Postoperative PROMs are presented in a variety of ways such as simple change in score reflecting a patients status at a given time point, the minimum clinically important difference (MCID), substantial clinical benefit (SCB), and patient acceptable symptom state (PASS)^13^. While healthcare professionals and researchers use these measures to interpret the significance of changes in PROMs and to compare the relative effectiveness of interventions, patients might not always understand their implications^14^. For example, if a patient has a high baseline pain or disability measure, they are more likely to achieve a threshold MCID change than a patient with a low baseline score (i.e. ceiling-floor effect). However, that same patient is more likely to end up with persistent moderate or severe pain and/or disability at a given post-intervention time point (i.e. status score)^15^. If looking at MCID achievement alone, this patient would be reported as a success. An alternate method of comparison of HRQoL is to determine the degree to which an individual, group or population is below or above the average for their country, age or sex^16^. Although most commonly used to assess the relative impact of a disease(s) on HRQoL, comparison to population normative data is also useful for the broader comparison of the relative benefit of interventions for a given disease or across various disease(s); between centres in a region, country or across countries^17^. Importantly, it is inherently easier for a patient to contextualize their HRQoL status in relation to the average HRQoL of their “normative” peers.

In this study, our primary objective was to compare the baseline and 1-year post-operative HRQoL outcomes of patients having spinal surgery for common degenerative conditions and to contextualize these HRQoL scores against normative age and sex matched data from the Canadian general population (CGP). Our secondary objectives were to review HRQoL outcomes by demographic and pathoanatomical subgroups and report on the portion of patients achieving the minimum clinically important difference (MCID) in physical function related HRQoL outcomes. Additionally, we assessed the HRQoL impact based on anatomical location (cervical vs. lumbar) and across the most common degenerative diagnoses for which spinal surgery was performed.

## Method

The Canadian Spine Outcomes and Research Network (CSORN) is a research initiative of the Canadian Spine Society with the intention to track and evaluate spine surgery outcomes across Canada. It currently consists of 22 participating sites across eight provinces. Before enrolling patients, Research & Ethics Board (REB) approval was obtained from the University of Toronto Health Network Research Ethics Board, as well as the REBs of all other participating sites (REB names approval numbers included in Supplementary Information File). Written informed consent was obtained from all participants and the study was conducted in accordance with the relevant guidelines. The registry collects hypothesis driven, comprehensive, longitudinal data (https://www.csorncss.ca). A wide variety of patient reported outcomes are collected as well as sociodemographic data. In addition, surgeons report diagnostic and clinical features, operative information and wait times^18^.

This study included prospectively enrolled patients who had degenerative cervical or lumbar pathology treated with elective spine surgery between January 1 st, 2015 and April 1 st, 2021. All patients were part of a national observational cohort study. Inclusion was based on a shared decision between the patient and treating surgeon to proceed with surgery and did not exclude patients based on any a priori clinical or surgical criteria. For this study, patients were excluded if they had a non-degenerative diagnosis, were undergoing revision surgery, or if they were missing baseline and 1-year HRQoL values. Adults over 25 years of age were selected to match the ages for which normative Short Form-12 (SF-12) data was available for the CGP^16^.

To match the currently available normative HRQoL data (see below), baseline and 1-year HRQoL outcomes were assessed via the SF-12 Physical Component Score (PCS) and Mental Component Score (MCS). The SF-12 PCS & MCS ranges from 0 to 100 with a higher score indicating better physical/mental function^19^. The proportion of patients achieving meaningful benefit from surgery was also examined. In published data from the CSORN registry and other jurisdictions, the mean baseline MCS for patient undergoing surgery for degenerative spinal condition is typically within a standard deviation of the normative value (50) with a significant, but relatively small degree of postoperative improvement compared to the PCS^20,21^. Consequently, our primary measure for pre- to post-operative change was limited to the PCS. A meaningful benefit was defined as^15^:.

1. Meeting or exceeding the Minimum Clinically Important Difference (MCID) values for the SF-12 PCS. To determine the appropriate MCID values, we performed a literature review with Preferred Reporting Items for Systematic Reviews and Meta-Analyses (PRISMA) guidelines. The PRISMA diagram and a summary of the 18 studies identified that calculated SF-12 PCS MCID values are presented in the Supplementary Information (Figure S1 and Table S1). A broad range of SF PCS MCID values has been reported with variation attributable to differences in the study population and MCID calculation technique used^15^. Studies examining patients with cervical myelopathy reported a range of SF PCS MCID values from 3.9 to 5.5^22–26^. Three of these five studies reported an MCID value between 3.9 and 4.1^22–24^. An SF PCS MCID value of 3.9 was selected for cervical myelopathy patients on the basis of the largest prospective study with a similar population to the present study^23^. A broader range (2.5–8.8) of SF PCS MCID values was described for lumbar conditions^27–36^. Eight of these ten studies were performed in a comparable North American population, however, all but two were single centre studies small cohort studies. A lumbar SF PCS MCID value of 4.9 was selected. This was derived from the remaining large single centre prospective and multicentre prospective studies^32,35^. Given the lack of high-quality data available from the literature, this value was also used for non-myelopathy cervical patients.
2. Patient satisfaction with surgery. A five-point Likert scale was used to determine patient satisfaction with surgery. Patients who responded “somewhat satisfied” or “extremely satisfied” were classified as satisfied with surgery.

Study reporting was done in concordance with the Strengthening the Reporting of Observational studies in Epidemiology (STROBE) guidelines^37^.

### Statistical analysis

To address the primary objective, we conducted a series of descriptive analyses. First, we compared changes from baseline to 1-year post-operative mean SF PCS and MCS values using paired t-tests. Second, we compared baseline and 1-year post-operative mean SF PCS and MCS values to those of the CGP. Normative CGP mean PCS and MCS were derived from values and standard deviations previously published for this population^16^. We used one sample t-test to compare PCS and MCS values with their corresponding CGP means (Table 1). Third, we reported the percentage of patients with PCS and MCS below the mean, below one standard deviation, and below two standard deviations of the CGP. For the secondary objective, we calculated the proportion of patients who had meaningful benefit from surgery, as well as the proportion of patients reporting being satisfied with the results of surgery. All analyses were conducted overall and stratified by age (25–64, > 65) and sex.

**Table 1:** Mean SF-36 PCS and MCS normative CGP values by age and sex subgroups. Data derived from Hopman et al. (2000).

Age	Male		Female		All	
	PCS	MCS	PCS	MCS	PCS	MCS
25–64	51.4	53	49.6	51.5	50.2	52
> 65	46.5	54.7	44.5	53.5	45.1	53.8
All (Mean)	51.4	52.6	49.7	50.9	50.5	51.7
All (SD)	*8.5*	*8.5*	*9.4*	*9.6*	*9.0*	*9.1*

We further conducted subgroup analyses by region (cervical or lumbar), primary diagnosis (degenerative disc disease [i.e. without instability or symptomatic root compression], disc herniation, stenosis, spondylolisthesis) and primary symptom for which surgery was being performed (myelopathy, radiculopathy, neurogenic claudication, and neck or low back pain).

All analyses were performed using SAS/STAT^®^ software version 9.4 (Copyright© 2020 SAS Institute Inc., Cary, NC, USA). A p-value of < 0.05 was taken as significant.

## Results

### Cohort selection and characteristics

**Fig. 1:**
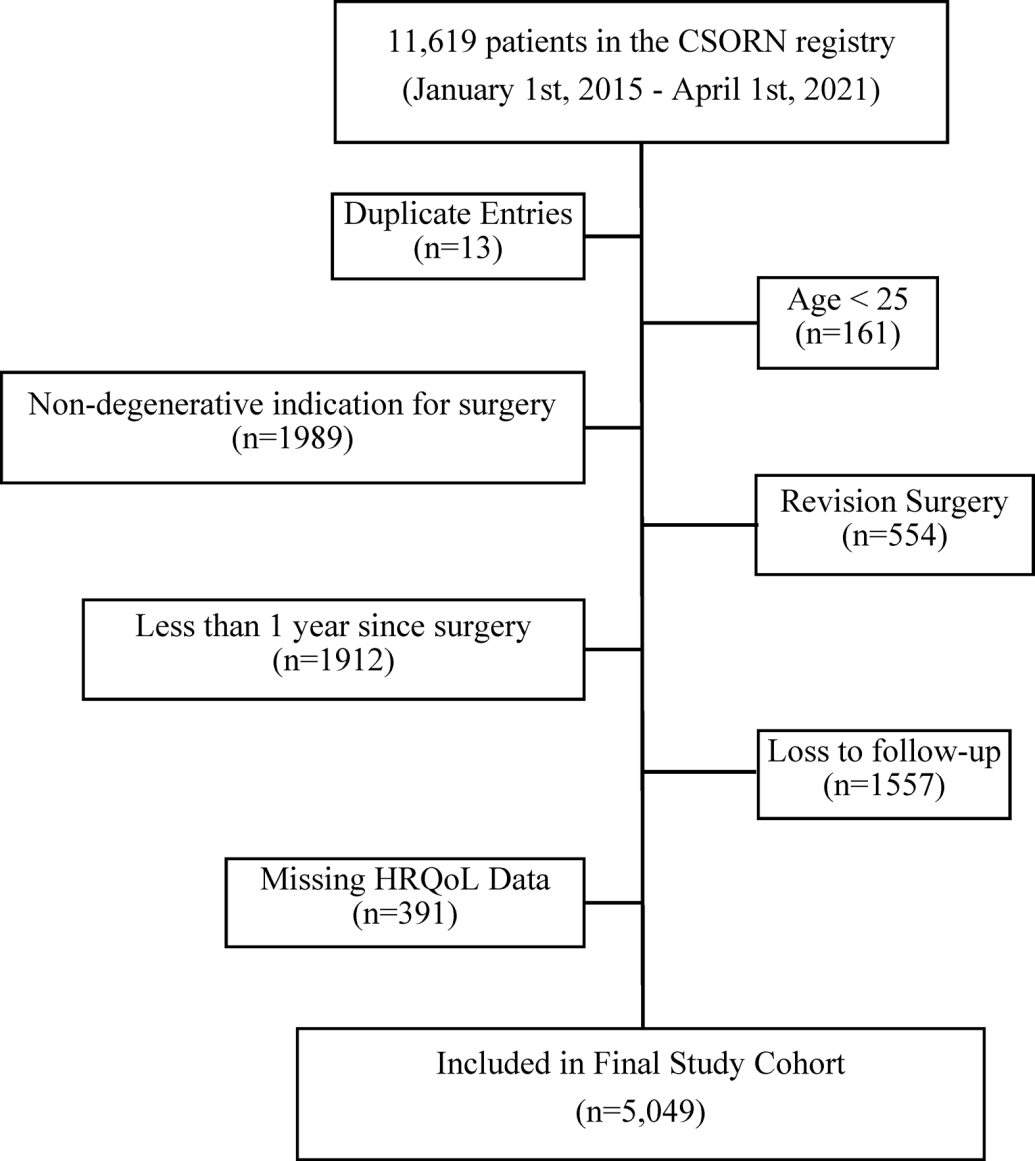
Flow diagram of the study cohort development. CSORN = The Canadian Spine Outcomes & Research Network. HRQoL = Short Form-12 Physical Component Score and Mental Component Score Health Related Quality of Life data.

Between January 1 st 2015 and April 1 st 2021, 11,619 patients were recorded in the CSORN database. The inclusion criteria were met by 6,997 patients, however, 1-year post-operative HRQoL data was incomplete in 391 patients and 1,557 patients were lost to follow-up, leaving 5049 patients (72.1%) in the final cohort (Fig. 1). The cohort demographics are summarised in Table 2. The mean age was 58.3 (SD 13.8), and the male: female ratio was 1.07. Most patients had lumbar surgery (84%). The most common primary symptom was radiculopathy (51%) followed by neurogenic claudication (29%) and axial back pain (12%). 8% of the cohort had surgery for cervical myelopathy. The most common pathoanatomical diagnosis was lumbar stenosis (32%) followed by spondylolisthesis and disc herniation (both 25%). 10% of the cohort had a diagnosis of degenerative disc disease.

**Table 2:** Cohort demographics.

	*n*	%
Total No. Patients	5049	
Mean Age	58.3	
25–64	3158	63
> 64	1891	37
Sex		
Male	2615	52
Female	2434	48
Surgery Site		
Cervical	833	16
Thoracolumbar	4216	84
Primary Symptom		
Axial Back Pain	597	12
Cervical Radiculopathy	407	8
Lumbar Radiculopathy	2175	43
Claudication	1444	29
Myelopathy	426	8
Pathoanatomical Diagnosis		
Degenerative Disc Disease	481	9
Disc Herniation	1245	25
Cervical Stenosis	458	9
Lumbar Stenosis	1603	32
Spondylolisthesis	1262	25

### Primary objective

**Fig. 2:**
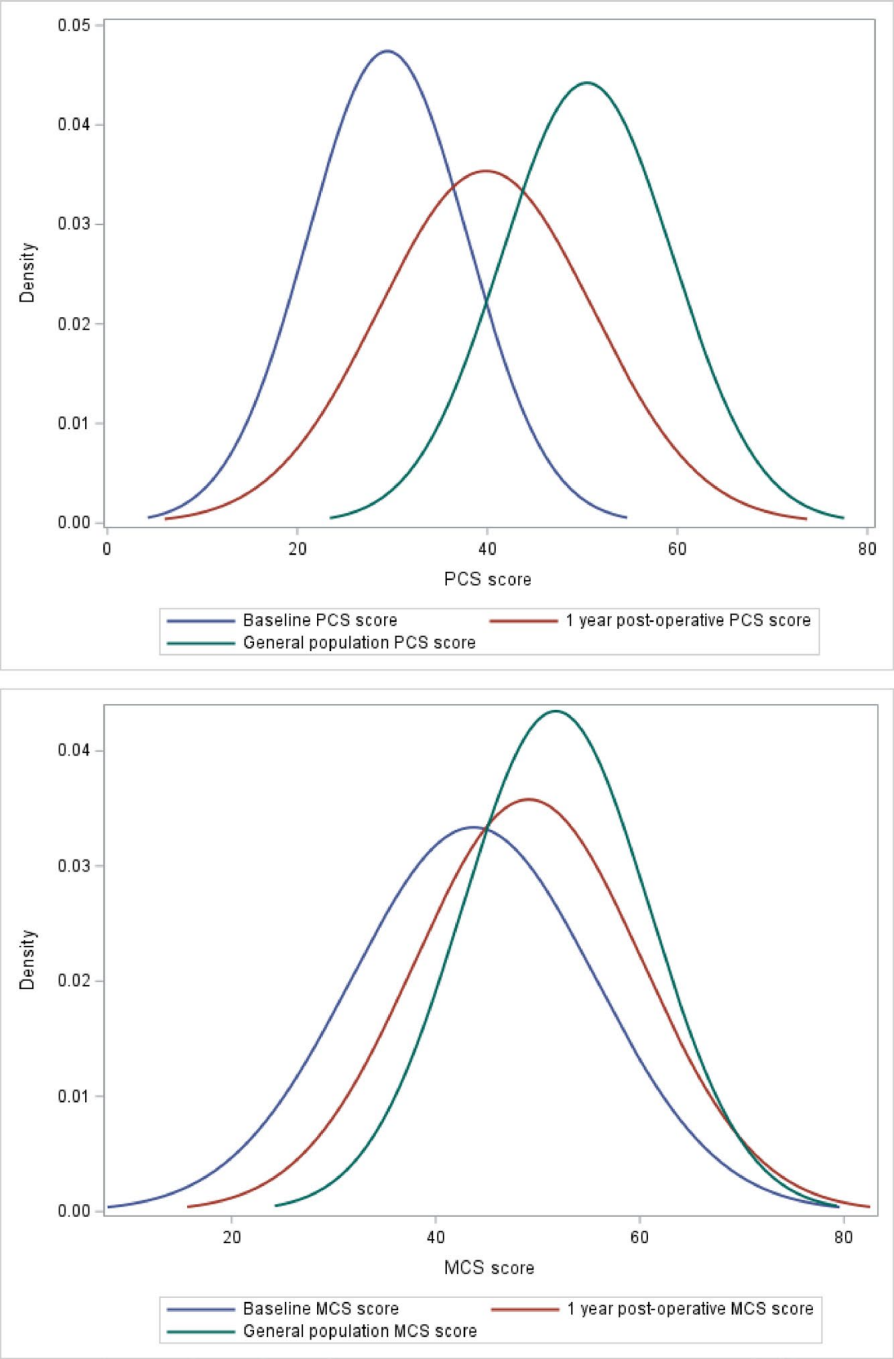
SF PCS & MCS histograms pre- and post-surgery compared to the normal Canadian population (25 + years).

Across the cohort, the mean pre-operative SF-12 PCS was 29.5 (SD 8.1) and MCS was 44.1 (SD 11.8). This improved to a mean PCS of 40.5 (SD 11.1, *p* < 0.0001) and MCS of 49.3 (SD 11.1, *p* < 0.0001) at the 1-year post-operative mark (Table 3). Histograms for the distribution of pre- and post-surgery PCS and MCS scores compared to the normal CGP (25 + years) are shown in Fig. 2. Overall, the mean baseline PCS was more than 2 SDs lower than the reported normative mean PCS of the CGP; this improved to being close to 1 SD from the normative CGP mean at 1-year after surgery^16^. Overall, the mean baseline MCS was within one standard deviation of the CGP normative mean and improved to being close to the normative population mean at 1-year after surgery (Fig. 2).

**Table 3:** Baseline and 1-year post-operative mean SF PCS and MCS values compared to age and sex matched peers from the Canadian general population (CGP). One sample t-tests comparisons of pre- and post-operative PCS means and MCS means to the corresponding CGP values were all significant (*p* < 0.0001). Paired t-tests comparing changes pre- and post-operative PCS/MCS for the overall cohort and by age/sex (*p* < 0.0001). Normative CGP population SF-12 PCS and MCS values have been established by *Hopman et al.* (2000). Normative CGP mean PCS and MCS for age and sex subgroups have been derived from this data, standard deviations were not available for these subgroups^16^.

PCS		Cohort			Male			Female		
		Baseline	1-YR	CGP	Baseline	1-YR	CGP	Baseline	1-YR	CGP
All	Mean	29.5	40.5	50.5	30.0	40.8	51.4	29.0	40.2	49.7
	Std. Dev	8.1	11.1	9.0	8.4	10.7	8.5	7.8	11.4	9.4
25–64	Mean	29.9	41.7	50.2	30.2	41.9	51.4	29.6	41.5	49.6
	Std. Dev	8.2	11.3		8.4	11.0		8.0	11.5	
> 65	Mean	28.7	38.5	45.1	29.6	39.0	46.5	27.8	38.0	44.5
	Std. Dev	7.9	10.5		8.3	10.2		7.4	10.9	

**MCS**		**Cohort**			**Male**			**Female**		
		**Baseline**	**1-YR**	**CGP**	**Baseline**	**1-YR**	**CGP**	**Baseline**	**1-YR**	**CGP**
All	Mean	44.1	49.3	51.7	45.1	49.8	52.6	43.0	48.8	50.9
	Std. Dev	11.8	11.1	9.1	11.8	10.7	8.5	11.7	11.4	9.6
25–64	Mean	42.6	48.4	52.0	43.6	49.0	53.0	41.6	47.8	51.5
	Std. Dev	11.7	11.3		11.7	11.0		11.5	11.5	
> 65	Mean	46.6	50.8	53.8	47.6	51.1	54.7	45.4	50.5	53.5
	Std. Dev	11.7	10.5		11.7	10.2		11.6	10.9	

**Fig. 3:**
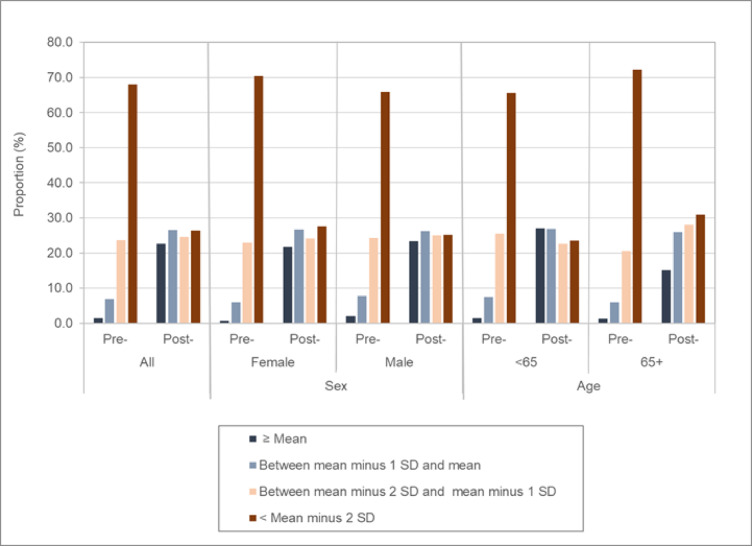
Percentage of patients with PCS at or above the CGP mean, within 1 SD below the mean, more than 1 but less than 2 SDs below the mean, and more than 2SDs below the mean. Values are provided pre- and post-surgery, and by age and sex subgroups.

The proportions of patients relative to the GCP mean, and 1–2 SDs below the mean are presented in Supplementary Table S2 and Fig. 3. Prior to surgery, 68.1% of patients are more than 2 SDs below the mean PCS scores of their peers in the CGP. One year after surgery, 22.6% of patients were at or above the CGP mean, 26.5% were within 1 SD, 24.5% were more than 1 SD but less than 2 SDs below the CGP mean, and 26.3% of patients remained more than 2 SDs below the CGP mean.

**Fig. 4:**
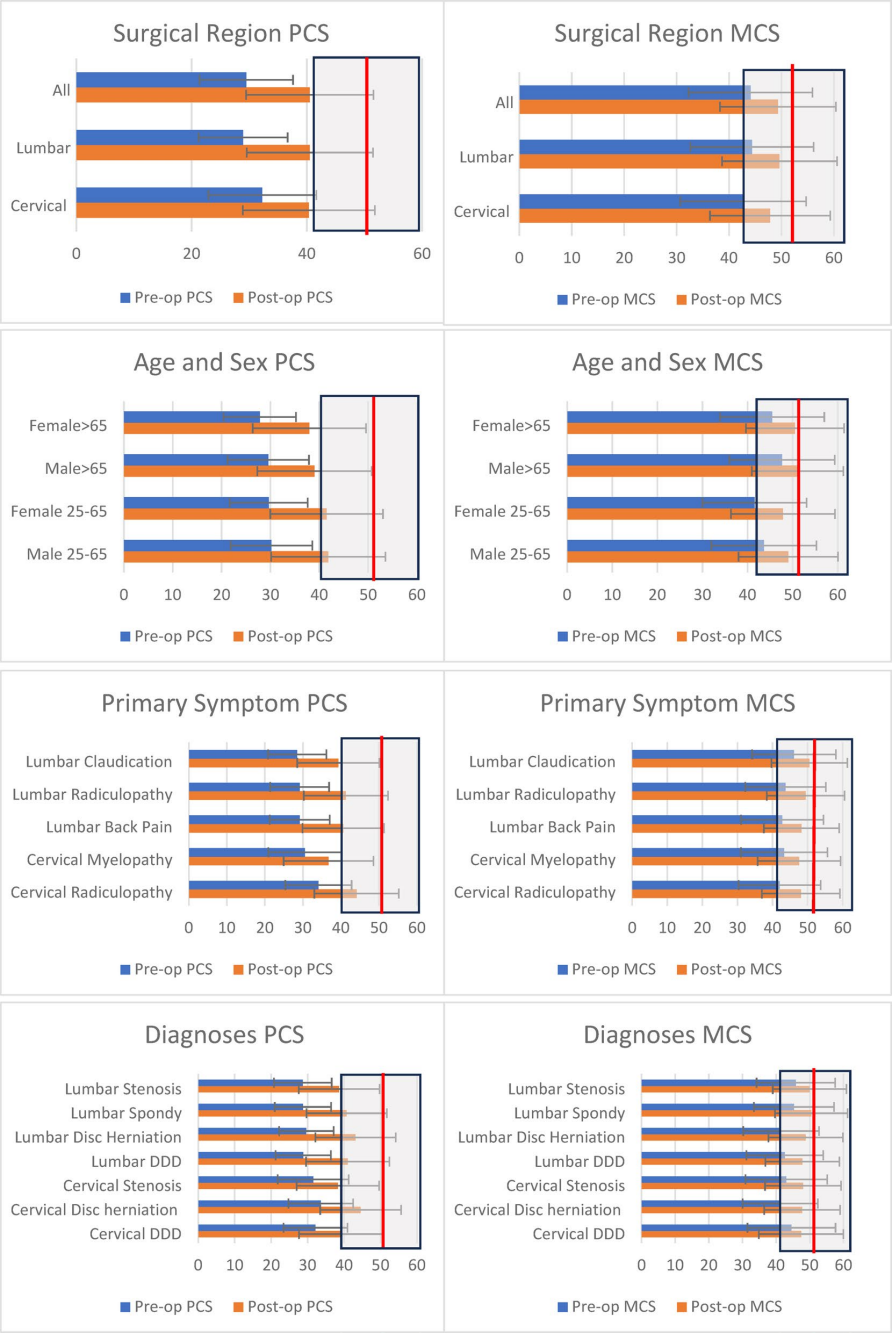
Charts depict the pre-op and post-op PCS and MCS by surgical region, age and sex, primary symptom, and diagnoses with the standard deviation provided as an error bar. The CGP mean is provided as the vertical red line for PCS and MCS respectively with the CGP standard deviation as a transparent grey box. Spondy = spondylolithesis, DDD = degenerative disc disease.

The mean baseline and 1-year post-operative PCS and MCS for defined subgroups relative to the mean of the CPG are shown in Fig. 4; Table 3. Subgroup comparisons demonstrated significant (*p* < 0.0001) improvement of PCS in all groups, with the greatest PCS change from baseline to 1-year post-operative for patients with lumbar disc herniation (Δ13.3)/radiculopathy (Δ12), and least for patients with cervical stenosis (Δ6.7)/myelopathy (Δ6.1).

### Secondary objective

Supplementary table S3 presents the proportion of patients achieving MCID by age-sex and principal pathology. Generally, differences in MCID achievement were small between men and women, but greater between age groups age (< 65 vs. 65+). Larger variability was found across diagnoses. For example, the percentage of patients achieving a PCS MCID of 4.9 was similar for disc herniation (75.5%), spondylolisthesis (72.9%), and degenerative disc disease (73.3%). The percentage of patients achieving the PCS MCID for lumbar stenosis was slightly lower, at 65.8%. A PCS MCID of 3.9 was achieved in 58.7% of patients with degenerative cervical myelopathy (DCM). DCM patients in the younger age group and female patients more commonly achieved the PCS MCID (61.9 vs. 53.4% and 64.6 vs. 55.1% respectively). In non-DCM cervical patients, a PCS MCID of 4.9 was achieved in 69.7% of patients having surgery for disc herniation, 58.5% of patients with DDD, and 67.3% of patients with a diagnosis of cervical radiculopathy.

Patient satisfaction with surgery was 85.5% for cervical procedures and 84.2% for lumbar procedures. The satisfaction rate ranged from 91.4% for cervical disc herniation to 81.3% for lumbar stenosis. Across all cervical diagnoses, younger patients were more satisfied than older patients with cervical procedures (86.8 vs. 80.9%). This was primarily due to a greater satisfaction rate in younger patients undergoing surgery for cervical radiculopathy (89.7% vs. 77.8%). There was a similar satisfaction rate amongst younger and older patients having surgery for DCM (82.6 vs. 81.4%) and surgery for lumbar conditions (84.6 vs. 83.5%). Patient satisfaction rates are summarised in Table 4. Furthermore, we found that satisfaction rates were significantly higher among those achieving MCID compared to those who did not (92.4% vs. 66.1% respectively). These findings were similar by spine location and diagnoses (Data not shown).

**Table 4:** Patient satisfaction rates by (A) diagnosis and age; and (B) primary symptom and age. DDD = Degenerative disc disease.

A.	All Cervical	DDD	Disc Herniation	Stenosis	All Lumbar	DDD	Disc Herniation	Spondylo-listhesis	Stenosis
All	85.5	84.7	91.4	82.3	84.2	83.7	85.6	86.8	81.3
25–64	86.8	84.9	91.7	83.6	84.6	84.2	86.1	86.5	80.9
≥ 65	80.9	84	87.5	79.6	83.5	81.4	81.6	87.3	81.5

B.	Cervical				Lumbar				
	**Radiculopathy**		**Myelopathy**		**Back Pain**		**Radiculopathy**		**Claudication**
**All**	**88.9**		**82.2**		**81.7**		**84.3**		**85**
25–64	89.7		82.6		82.5		84.6		86.6
≥ 65	77.8		81.4		79.1		83.6		84.1

## Discussion

This observational study prospectively evaluated a national surgical cohort that is representative of the most common degenerative spinal conditions receiving surgery. Our study demonstrates that patients undergoing surgical intervention for degenerative spinal conditions preoperatively report a profoundly reduced physical HRQoL (more than two standard deviations below) compared to age and sex matched peers in the CGP. One-year following spine surgery, approximately 1 in 4 patients physical HRQoL were above or at the mean CGP (22.6%), within 1 SD below the mean (26.5%), more than 1 but less than 2 SDs below the mean (24.6%), or remained more than 2 standard deviations below the CPG mean (26.3%). However, the degree of improvement relative to the CGP PCS score was less in older patients (65+) and those presenting with degenerative cervical myelopathy. Preoperative MCS scores were typically at or within one standard deviation of the mean MCS for the CGP and improved at one-year post-surgery across the overall cohort and in all subgroups. For our secondary objectives, MCID for PCS was achieved by the majority of patient and overall demonstrated relatively minimal variation between men and women, but greater variation across diagnoses and age (< 65 vs. 65+) within diagnoses.

Our results are consistent with the literature on the impact of spinal surgery on HRQoL^3,4,9–11^. Nayak et al. performed a meta-analysis of 99 studies published between 2000 and 2014 and reported that post-operative HRQoL scores improved across all groups following spine surgery, with a range of 8.08–15.25 point improvement in SF PCS^3^. Similar to the present study, the largest change was seen in patients with lumbar radiculopathy, while the smallest PCS change was seen in patients with cervical myelopathy. To the best of our knowledge, our study is the largest to compare spine surgery HRQoL impact to normative population data. In a limited consecutive series of 100 patients, Mokhtar et al. reported that spinal fusion for degenerative lumbar spondylolisthesis in an Australian population was effective in returning SF PCS scores to closer to the normative population mean to a similar degree demonstrated in the current study^4^. The relatively high baseline MCS scores of our entire cohort most likely reflects an underlying surgical patient selection bias related to the known negative prognostic implications of poor baseline mental health on surgical outcomes^38^. However, Hopman et al., have demonstrated that mental health scores may remain high despite the negative effects of different chronic illness on physical health^39^.

Our results suggest that the capacity for functional improvement in patient with DCM is not as great as for those with other degenerative spinal conditions. This is most likely attributable to the fundamental difference in the impact of compressive disease on the spinal cord versus the spinal nerve roots rather than to the effect of surgery^40^. In degenerative cervical myelopathy, loss of physical function is attributable to neurological dysfunction and surgery is effective in preventing further impairment due to disease progression but may not always result in functional improvement^41^. The neurological/surgical urgency of DCM is reflected through the mean surgical wait times in our cohort. Patients with DCM had surgery substantially sooner than those with pain dominant conditions (mean of 68 days compared to 121 to 176 days for the other diagnoses, Supplementary Table S4). In our study, the mean PCS scores of patients with DCM still improved from over two standard deviations below the normative population mean to between 1 and 2 standard deviations away from this mean after surgery (30.5 to 36.8 respectively). This 6.3 point improvement in PCS is concordant with the 6.02 point improvement reported in a large, multicentre North American prospective study^23^. Karim et al. have also examined SF PCS outcomes in DCM patients stratified by disease severity. Whilst patients with mild, moderate, and severe disease all demonstrated improvements in SF PCS 12-months after surgery, the percentage of patients achieving the PCS MCID is lower for patients with moderate or severe disease than it is for mild disease, imparting the impact of established neurological injury on physical function^42^.

Conversely, in the other symptom presentation and associated pathoanatomical diagnoses not causing myelopathy, pain is the dominant cause of functional limitation. Relative to neurological dysfunction, pain dominant conditions appear to have a greater capacity for physical functional improvement after surgery^43^. The relatively lower proportion of patients achieving the PCS MCID with spinal stenosis compared to radiculopathy or DDD is likely related to two factors. Firstly, radiculopathy and DDD patients are typically 10–20 years younger and have relatively focal spinal disease and surgery compared to patients with spinal stenosis^3^. Secondly, the principal pathology causing spinal stenosis is osteoarthritis of the facet joints which is commonly associated with a high degree of other symptomatic appendicular joints that can negatively affect overall reported HRQoL. Perruccio et al., has demonstrated that patients undergoing surgery for lumbar spinal stenosis reported 1+ (77%), 2+ (63%), and 4+ (25%) symptomatic joint sites other than their low back. Increasing symptomatic joints was associated with increasing risk of not achieving a MCID (odds ratio [OR]: 1.32, 95% confidence interval [CI]: 1.05, 1.66)^44^.

The success of total hip arthroplasty (THA) and total knee arthroplasty (TKA) have become the gold standards for orthopaedic intervention due to their cost-effective impact in reducing pain, improving function and quality of life^45^. Prior studies have reported that patients undergoing lumbar spinal surgery demonstrate comparable improvements in HRQoL to those undergoing total joint arthroplasty^4,9–11,45^. Rampersaud et al. reported that total joint arthroplasty PCS scores improved from a starting point of over two standard deviations away from the population norm to being close to one standard deviation away post-operatively^16^. The current national study demonstrates that elective spine surgery across the most common degenerative conditions with functional limitation (SF-PCS) due to pain, achieves a similar positive impact on HRQoL. Our national study also demonstrates that surgical intervention for the most common spinal diagnoses resulted in meaningful improvement (MCID) in self-reported physical function (SF-PCS) for the majority of patients with an associated high satisfaction rate (85%). This degree of patient satisfaction is also similar to reported rates following THA/TKA reported in Canada (93%-THA and 88%-TKA) and the UK (86.6%)^46,47^.

There are several limitations to the study. Our data are limited to Canadian patients within a single payer healthcare system and may not be generalizable to other health jurisdictions. However, our patient reported findings are similar to reports from other first world health settings with varied regional and national healthcare delivery models and payer systems.^3,4, 36, 37^ Given the large number of surgeons and institutions involved, the surgical treatment of patients for a given pathology was not standardized and was at the discretion of the attending surgeon. We believe that the inclusion of this practice variation makes our findings more reflective of real-world practice, where there is often more than one surgical approach available. The PCS MCID values may vary depending on factors such as the study population, underlying spine pathology, method of treatment, sample size and patient characteristics, so the proportion of our patients achieving a clinically meaningful improvement in HRQoL may not be generalizable^35^. However, a review of the relevant international literature for similar surgical populations informed the most appropriate MCID threshold values used in our study. The assessment of HRQoL at the one-year post-operative mark is only indicative of short term post-surgical outcome, however, data from previous studies suggests good durability of these outcomes in the longer term^48–51^.

Finally, one-year HRQoL data was not available for 28% of the eligible cohort, representing a potential source of bias. To further explore this, an analysis of the baseline characteristics of included patients compared to those lost to follow-up was performed (Supplemental Table S5). Importantly, there was no baseline difference in the primary outcome measure, mean SF PCS between included and lost to follow-up groups. As result of the large cohort size, there were several statistically significant differences in the baseline demographics between the two groups (age, sex, surgery site, primary symptom and pathoanatomical diagnosis, as well as ODI). However, these differences were small and not clinically meaningful. Patients in the lost to follow-up group were more likely to be younger (< 45), male, have a primary symptom of radiculopathy and have a diagnosis of disc herniation. We note that this subset of patients has a particularly high satisfaction rate with surgery which may contribute to difficulty with longer term follow-up. Loss to follow-up is an inherent issue with registry data and our 1-year follow-up rates are similar to those expected and reported in other national spine registries^52–54^, as well as joint arthroplasty registries collecting HRQoL data^55,56^. In a review of spine registry data aiming to provide recommendations to improve the quality of evidence from registries, a 60–80% 1-year follow-up response rate was recommended^52^. The 1-year response rate in our study was 72.1%, falling within this recommended range. Additionally, several spine registry sub-population studies have noted their lost to follow-rate may not bias outcomes^57–59^, none the less attrition bias remains a pertinent limitation.

The results of our study aid both spinal surgeons and other physicians with patient counselling. They contextualise improvements in physical function HRQoL outcomes against the average of the CGP, as well as their age and sex matched peers, making it simpler for physicians and patients to conceptualise surgical outcomes compared to reporting success based on MCIDs.

## Conclusion

Preoperatively, patients with most common degenerative spinal conditions report profound impairment of their physical function HRQoL scores compared to age and sex matched peers in the CGP. Our study demonstrates that spinal surgery for these pathologies is effective in improving physical HRQoL in the majority of patients, but typically not to the mean CGP norms. The degree of physical function HRQoL improvements was less in older patients (65+) and those presenting with degenerative cervical myelopathy. The results of this study provide national level data to aid patient perioperative patient counselling regarding more realistic HRQoL relative to the national average. Future work will examine physical HRQoL changes between comparable patients within the CSORN spinal surgery registry and the Canadian National Hip & Knee arthroplasty registries.

## Electronic supplementary material

Below is the link to the electronic supplementary material.

Supplementary Material 1

## Data Availability

Data availability statementThe datasets generated during and/or analysed during the current study are not publicly available due to legal and ethical restrictions but may be available from the Canadian Spine Outcomes and Research Network for researchers meeting the criteria for access to confidential data. Interested parties can contact CSORN (email gmcintosh@spinecanada.ca), to facilitate requests.
