# Supplementary material for: The impact of elective spine surgery in Canada for degenerative conditions on patient reported health-related quality of life outcomes
Source: Sci Rep. 2025 May 31;15:19143. doi: 10.1038/s41598-025-03613-4 (PMC12126512; doi:10.1038/s41598-025-03613-4)
Supplement: Supplementary file 1 — Supplementary Material 1 [file 41598_2025_3613_MOESM1_ESM.docx]

# Supplementary Information

**Identification of studies via databases and registers**

Records removed *before screening*:

Duplicate records removed (n = 0)

Records marked as ineligible by automation tools (n = 0)

Records removed for other reasons (n = 0)

Records identified from*:

Databases (n = 143)

**Identification**

Records screened

(n = 143)

Records excluded**

(n = 121)

Reports sought for retrieval

(n = 22)

Reports not retrieved

(n = 0)

**Screening**

Reports assessed for eligibility

(n = 22)

Reports excluded:

Did not calculate SF 8/12/36 PCS in spine surgery patients (n = 4)

Studies included in review

(n = 18)

**Included**

**Figure S1**. PRISMA diagram of literature search for SF PCS MCID values.

**Table S1.** Summary of 18 studies that calculated SF PCS MCID values.

| **Author** | **Year** | **Diagnosis** | **Procedure** | **Study Population** | **No. Patients** | **SF 12/36** | **MCID Calculation Methods** | **Final Method** | **SF PCS MCID** | **MCID Achieved** |
| --- | --- | --- | --- | --- | --- | --- | --- | --- | --- | --- |
| Nie et al.^25^ | 2023 | Mixed | ALIF | Single centre retrospective (USA) | 32 | SF 12 | Anchor & Distribution | MDC | 8.2 | 53.10% |
| Nie et al.^23^ | 2023 | Mixed | MIS-TLIF | Single centre retrospective (USA) | 71 | SF 12 | Anchor & Distribution | ROC | 6.8 | 54.90% |
| Nie et al.^24^ | 2023 | Mixed | LLIF | Single centre retrospective (USA) | 26 | SF 12 | Anchor & Distribution | ROC Closest to (0,1) | 6.4 | 61.50% |
| Nakarai et al.^26^ | 2022 | LSS | Decompression only | Multicentre Retrospective (Japan) | 422 | SF 12 | Anchor | ROC % change | 13.70%* | 56.90% |
| Ogura et al.^27^ | 2020 | LSS | Decompression only | Single centre retrospective (Japan) | 126 | SF 8 | Anchor & Distribution | Anchor | 4.77 | NR |
| Kato et al.^18^ | 2019 | Cervical myelopathy | Cervical laminoplasty | Multicentre Retrospective (Japan) | 101 | SF 36 | Anchor & Distribution | ROC | 3.9 | NR |
| Badhiwala et al.^19^ | 2018 | Cervical myelopathy | Mixed | Multicentre Prospective (North America) | 606 | SF 36 | Anchor & Distribution | ROC | 3.93 | 55.20% |
| Zhou et al.^20^ | 2015 | Cervical myelopathy | Mixed | Single centre retrospective (China) | 113 | SF 36 | Anchor & Distribution | ROC | 4.09 | NR |
| Zhang et al.^21^ | 2015 | Cervical myelopathy | Mixed | Single centre prospective (China) | 140 | SF 36 | Distribution | 1-SEM | 5.5 | NR |
| Auffinger et al.^43^ | 2013 | Mixed cervical | Mixed | Single centre prospective (USA) | 88 | SF 36 | Anchor & Distribution | MDC | 5.6 | NR |
| Auffinger et al.^22^ | 2013 | Cervical myelopathy | ACDF | Single centre prospective (USA) | 29 | SF 36 | Anchor & Distribution | MDC | 5.56 | NR |
| Parker et al.^44^ | 2013 | Cervical radiculopathy | ACDF | Single centre prospective (USA) | 61 | SF 12 | Anchor | MDC | 8.1 | NR |
| Carreon et al.^28^ | 2013 | Mixed lumbar | Decompression and fusion | Single centre prospective (USA) | 1055 | SF 36 | Anchor | MDC | 4.9-5.21 | NR |
| Parker et al.^32^ | 2012 | Lumbar pseudoarthrosis | Revision lumbar fusion | Single centre retrospective (USA) | 47 | SF 12 | Anchor | Change difference | 4.4 | NR |
| Parker et al.^30^ | 2012 | Recurrent lumbar stenosis | Revision decompression and fusion | Single centre retrospective (USA) | 53 | SF 12 | Anchor | MDC | 2.5 | NR |
| Parker et al.^29^ | 2012 | Lumbar ASD | Revision lumbar fusion | Single centre retrospective (USA) | 50 | SF 12 | Anchor | MDC | 8.8 | NR |
| Carreon et al.^45^ | 2010 | Mixed cervical | Cervical fusion | Single centre prospective (USA) | 505 | SF 36 | Anchor & Distribution | ROC | 4.1 | NR |
| Copay et al.^31^ | 2008 | Mixed lumbar | Mixed lumbar decompression +/- fusion | Multicentre Prospective (USA) | 454 | SF 36 | Anchor & Distribution | MDC | 4.9 | 47.80% |

Abbreviations: LSS = Lumbar spinal stenosis, ASD = adjacent segment disease, ALIF = anterior lumbar interbody fusion, MIS-TLIF = minimally invasive transforaminal lumbar interbody fusion, LLIF = lateral interbody fusion, ACDF = anterior cervical discectomy and fusion, SF = Short Form 12/36, MDC = minimum detectable change, ROC = receiver operator characteristics, SEM = standard error of measurement, NR = not reported.

* Calculated MCID as a percentage improvement on baseline

**Table S2.** Percentage of patients with PCS and MCS at or above the CGP mean, between the CGP mean and mean minus 1SD, between mean minus 1SD and mean minus 2SD. Values are provided pre- and post-surgery, and by age-sex subgroups.

| **PCS** | | | | | | | | |
| --- | --- | --- | --- | --- | --- | --- | --- | --- |
|  | **≥ Mean** | | **Between mean minus 1 SD and mean** | | **Between mean minus 2 SD and  mean minus 1 SD** | | **< Mean minus 2 SD** | |
|  | **Pre** | **Post** | **Pre** | **Post** | **Pre** | **Post** | **Pre** | **Post** |
| Overall |  |  |  |  |  |  |  |  |
| All | 1.4 | 22.6 | 6.9 | 26.5 | 23.6 | 24.6 | 68.1 | 26.3 |
| <65 | 1.5 | 27.0 | 7.4 | 26.8 | 25.5 | 22.6 | 65.6 | 23.6 |
| 65+ | 1.3 | 15.1 | 6.0 | 26.0 | 20.6 | 28.0 | 72.1 | 30.9 |
| Female |  |  |  |  |  |  |  |  |
| All | 0.8 | 21.7 | 5.9 | 26.7 | 22.9 | 24.1 | 70.5 | 27.5 |
| <65 | 1.0 | 26.6 | 6.8 | 26.9 | 25.0 | 22.5 | 67.2 | 24.0 |
| 65+ | 0.3 | 13.2 | 4.4 | 26.4 | 19.2 | 26.8 | 76.1 | 33.6 |
| Male |  |  |  |  |  |  |  |  |
| All | 2.0 | 23.4 | 7.8 | 26.3 | 24.3 | 25.1 | 65.9 | 25.2 |
| <65 | 1.9 | 27.5 | 8.0 | 26.7 | 25.9 | 22.6 | 64.2 | 23.2 |
| 65+ | 2.1 | 16.8 | 7.5 | 25.5 | 21.8 | 29.1 | 68.7 | 28.5 |
| **MCS** | | | | | | | | |
| Overall |  |  |  |  |  |  |  |  |
| All | 28.1 | 49.0 | 26.5 | 25.4 | 25.9 | 15.7 | 19.5 | 9.9 |
| <65 | 23.3 | 46.4 | 26.0 | 25.2 | 27.7 | 17.1 | 22.9 | 11.4 |
| 65+ | 36.0 | 40.8 | 27.3 | 38.1 | 23.0 | 32.2 | 13.8 | 28.0 |
| Female |  |  |  |  |  |  |  |  |
| All | 24.9 | 47.1 | 25.5 | 25.1 | 27.2 | 17.0 | 22.4 | 10.8 |
| <65 | 20.7 | 43.8 | 24.1 | 25.4 | 28.6 | 18.4 | 26.6 | 12.4 |
| 65+ | 32.2 | 52.8 | 27.8 | 24.6 | 24.8 | 14.6 | 15.3 | 8.0 |
| Male |  |  |  |  |  |  |  |  |
| All | 31.0 | 50.7 | 27.5 | 25.7 | 24.8 | 14.5 | 16.8 | 9.1 |
| <65 | 25.9 | 48.7 | 27.8 | 25.0 | 26.8 | 15.8 | 19.5 | 10.5 |
| 65+ | 39.4 | 53.8 | 26.8 | 26.8 | 21.4 | 12.5 | 12.4 | 6.9 |

**Table S3.** MCID achievement (%) by age, sex, and pathology

|  |  | **Overall** | **Women** | **Men** |
| --- | --- | --- | --- | --- |
| **All pathologies** |  |  |  |  |
| Overall |  | 5049 (69.0%) | 2434 (69.8%) | 2615 (68.3%) |
| <65 years |  | 3158 (71.1%) | 1538 (71.6%) | 1619 (70.7%) |
| 65+ years |  | 1891 (65.4%) | 896 (66.6%) | 996 (64.3%) |
| **Degenerative cervical myelopathy** |  |  |  |  |
| Overall |  | 426 (58.7%) | 159 (64.6%) | 267 (55.1%) |
| <65 years |  | 265 (61.9%) | 103 (59.8%) | 162 (63.4%) |
| 65+ years |  | 161 (53.4%) | 56 (73.2%) | 105 (42.3%) |
| **Degenerative disc disease** |  |  |  |  |
| Overall |  | 481 (73.3%) | 229 (70.1%) | 252 (73.1%) |
| <65 years |  | 405 (72.7%) | 191 (70.1%) | 214 (75.0%) |
| 65+ years |  | 76 (66.1%) | 38 (69.7%) | 38 (62.5%) |
| **Disc herniation** |  |  |  |  |
| Overall |  | 1245 (75.1%) | 586 (74.9%) | 659 (75.3%) |
| <65 years |  | 1131 (75.1%) | 535 (74.7%) | 596 (75.5%) |
| 65+ years |  | 114 (75.2%) | 51 (77.6%) | 63 (73.3%) |
| **Spondylolisthesis** |  |  |  |  |
| Overall |  | 1262 (72.9%) | 800 (74.0%) | 462 (70.6%) |
| <65 years |  | 682 (75.3%) | 433 (78.4%) | 249 (70.1%) |
| 65+ years |  | 580 (69.8%) | 367 (68.9%) | 213 (71.3%) |
| **Cervical stenosis** |  |  |  |  |
| Overall |  | 458 (62.9%) | 213 (61.4%) | 245 (64.2%) |
| <65 years |  | 412 (64.0%) | 185 (62.3%) | 227 (65.3%) |
| 65+ years |  | 46 (53.4%) | 28 (55.6%) | 18 (50.0%) |
| **Lumbar stenosis** |  |  |  |  |
| Overall |  | 1603 (65.8%) | 638 (63.5%) | 965 (66.8%) |
| <65 years |  | 642 (66.7%) | 259 (64.7%) | 383 (68.1%) |
| 65+ years |  | 961 (64.6%) | 379 (62.7%) | 582 (65.9%) |

**Table S4.** Wait time from decision to proceed with surgery to date of surgery by primary symptom.

| **Primary Symptom** | **N** | **Mean** |
| --- | --- | --- |
| **Axial Back Pain** | 510 | 175.9 |
| **Radiculopathy** | 2260 | 113.4 |
| **Myelopathy** | 353 | 67.7 |
| **Neurogenic Claudication** | 1262 | 128.2 |
| All | 4385 | 121.2 |

N = Number

**Table S5.** Statistical analysis of study cohort in comparison to lost to follow-up cohort.

|  | **Included** | **Loss to follow-up** | **Missing SF12** | **p-value** |
| --- | --- | --- | --- | --- |
| **Total No. Patients** | 5049 | 1557 | 391 |  |
| **Mean Age (SD)** | 58.3 (13.8) | 55.7 (14.5) | 60.6 (13.7) | <0.0001 |
| **Age groups** |  |  |  | <0.0001 |
| *25-64* | 62.5% | 68.9% | 58.1% |  |
| *>64* | 37.5% | 31.1% | 41.9% |  |
| **Sex** |  |  |  | 0.0103 |
| *Male* | 52.0% | 56.4% | 52.6% |  |
| *Female* | 48.0% | 43.6% | 47.4% |  |
| **Surgery Site** |  |  |  | 0.0273 |
| *Cervical* | 16.6% | 19.5% | 17.8% |  |
| *Thoracolumbar* | 83.4% | 80.5% | 82.2% |  |
| **Primary Symptom** |  |  |  | <0.0001 |
| *Axial Back Pain* | 11.9% | 9.4% | 10.0% |  |
| *Cervical Radiculopathy* | 8.1% | 7.6% | 10.3% |  |
| *Lumbar Radiculopathy* | 43.2% | 48.0% | 39.2% |  |
| *Claudication* | 8.4% | 11.9% | 7.5% |  |
| *Myelopathy* | 28.3% | 23.1% | 33.0% |  |
| **Pathoanatomical Diagnosis** |  |  |  | <0.0001 |
| *Degenerative Disc Disease* | 9.5% | 7.1% | 8.0% |  |
| *Disc Herniation* | 24.7% | 33.1% | 21.2% |  |
| *Cervical Stenosis* | 9.1% | 10.8% | 9.8% |  |
| *Lumbar Stenosis* | 31.9% | 29.0% | 36.7% |  |
| *Spondylolisthesis* | 24.7% | 20.1% | 24.4% |  |
| **Baseline PROMs (Mean (SD))** |  |  |  |  |
| *PCS* | 29.5 (8.1) | 29.5 (8.4) | 29.3 (7.9) | 0.8781 |
| *MCS* | 44.1 (11.8) | 42.7 (12.1) | 43.2 (13.1) | 0.0005 |
| *ODI/NDI* | 45.9 (15.7) | 47.4 (16.9) | 48.1 (16.1) | 0.0005 |
| *Pain* | 7.6 (1.9) | 7.7 (2.0) | 7.7 (1.8) | 0.3282 |
| *Comorbidities* | 2.9 (1.9) | 2.9 (1.8) | 3.0 (1.8) | 0.6726 |

**Participating Site Research & Ethics Board Approval Numbers**

Universite de Sherbrooke:

Registre Société Canadienne Rachis:

Registre SCR multi

REB approval numbers: CHUS 2016-1246, MP-20-2012-1643

Saint John: Horizon Health Network Research Ethics Board
Neuro: REB approval number: 22983

Ortho: REB approval number: 18700

University of Calgary: Conjoint Health Research Ethics Board
REB approval number: REB15-1332_REN7

L’enfant Jesus (Quebec), Montreal: Comite d'ethique de la recherche, CHU de Quebec – Universite Laval

REB approval number: F9-50062

McGill University Montreal: MUHC Centre for Applied Ethics

REB approval number: 2013-2106, 12-121-SDR

QEII Halifax: Nova Scotia Health Authority Research Ethics Board
Neuro: REB approval number: 1012957

Ortho: REB approval number: 1012049

### Sault Ste Marie: Joint Group Health Centre/Sault Area Hospital Research Ethics Board

REB approval number: 2017-04-01

St John’s: Newfoundland and Labrador Health Research Ethics Board (HREB)

Approved but no designated REB approval number used

Ottawa: Ottawa Health Science Network Research Ethics Board (OHSN-REB)

REB approval number: 20120539-01H

Sunnybrook Health Sciences Centre: Research Ethics Board of Sunnybrook Health Sciences Centre

REB approval number: PIN 2593

St Michael’s Hospital: Unity Health Toronto Research Ethics Board

REB approval number: 13-135

Toronto Western Hospital: University Health Network Research Ethics Board

REB approval number: 12-5190

University of Alberta (Edmonton): Health Research Ethics Board - Health Panel
REB approval number: Pro00036987_REN7

Vancouver General Hospital: UBC Clinical Research Ethics Board
REB approval number: H12-01627

Victoria BC: Vancouver Island Health Authority Health Research Ethics Board (HREB)

REB approval number: 101089-123194

Victoria Hospital, London: University of Western Ontario Research Ethics Board for Health Sciences Research Involving Human Subjects (HSREB)

REB approval number: 103079

Winnipeg Health Sciences: University of Manitoba Health Research Ethics Board

REB approval number: HS15352 (H2012:1
